# Surgical Treatment for Skeletal Metastases From Soft Tissue Sarcomas: Experience With 23 Lesions in 20 Patients

**DOI:** 10.1080/13577149878064

**Published:** 1998-06

**Authors:** Hideki Yoshikawa, Takafumi Ueda, Ikuo Kudawara, Nobuhito Araki, Kazuo Yonenobu, Takahiro Ochi, Atsumasa Uchida

**Affiliations:** 1 Department of Orthopaedic Surgery Osaka Medical Center for Cancer and Cardiovascular Diseases 1–3–3, Nakamichi Higashinari-ku Osaka 537 Japan; 2 Department of Orthopaedic Surgery Osaka University Medical School Suita Japan; 3 Department of Orthopaedic Surgery Mie University Medical School Tsu Japan

## Abstract

*Purpose.* This paper reports the procedures and the clinical results of a series of surgical treatments for skeletal metastases from soft tissue sarcomas.

*Subjects and methods.* Surgical treatment of metastatic bony lesions from soft tissue sarcomas has been carried out over a 20 year period (1975–1996). Thirty-two patients developed skeletal metastases from soft tissue sarcomas, and 20 of these cases received surgical treatment. The 23 metastatic bony lesions in these 20 patients were treated using the following surgical approaches: wide resection with prosthetic replacement in five lesions, wide or marginal resection without reconstruction in four lesions, intramedullarly nailing with curettage and methylmethacrylate cementation in four lesions, marginal resection of vertebral body with replacement by a ceramic prosthesis in three lesions, laminectomy in three lesions, intramedullarly nailing in two lesions, and curettage in two lesions.

*Results.* Relief of pain was achieved in 17 of the 20 patients. The ambulatory status of the patients with metastasis in the lower extremity or periacetabular region was significantly improved in nine of 10 cases. Seventeen patients died of disease, with a mean survival period of 17.9 months after surgery for metastasis.

*Discussion.* Although surgical treatment for skeletal metastases from soft tissue sarcomas cannot save the life of the patient, it can be of value in improving their well-being and overall quality of life. In these cases, surgical intervention may be more frequently indicated than in tumors with an osteoblastic or mixed pattern.


Sarcoma (1998) 2, 107–114

ORIGINAL ARTICLE

### Surgical treatment for skeletal metastases from soft tissue sarcomas: experience with 23 lesions in 20 patients

HIDEKI YOSHIKAWA,[1] TAKAFUMI UEDA,[1] IKUO KUDAWARA,[1] NOBUHITO ARAKI,[2] KAZUO YONENOBU,[2] TAKAHIRO OCHI[2] & ATSUMASA UCHIDA[3]

[1]*Department of Orthopaedic Surgery, Osaka Medical Center for Cancer and Cardiovascular Diseases, Osaka, Japan &* [2]*Department of Orthopaedic Surgery, Osaka University Medical School, Suita, Japan &* [3]*Department of Orthopaedic Surgery, Mie University Medical School, Tsu, Japan*

**Abstract**

*Purpose.* This paper reports the procedures and the clinical results of a series of surgical treatments for skeletal metastases from soft tissue sarcomas.

*Subjects and methods.* Surgical treatment of metastatic bony lesions from soft tissue sarcomas has been carried out over a 20 year period (1975–1996). Thirty-two patients developed skeletal metastases from soft tissue sarcomas, and 20 of these cases received surgical treatment. The 23 metastatic bony lesions in these 20 patients were treated using the following surgical approaches: wide resection with prosthetic replacement in five lesions, wide or marginal resection without reconstruction in four lesions, intramedullarly nailing with curettage and methylmethacrylate cementation in four lesions, marginal resection of vertebral body with replacement by a ceramic prosthesis in three lesions, laminectomy in three lesions, intramedullarly nailing in two lesions, and curettage in two lesions.

*Results.* Relief of pain was achieved in 17 of the 20 patients. The ambulatory status of the patients with metastasis in the lower extremity or periacetabular region was significantly improved in nine of 10 cases. Seventeen patients died of disease, with a mean survival period of 17.9 months after surgery for metastasis.

*Discussion.* Although surgical treatment for skeletal metastases from soft tissue sarcomas cannot save the life of the patient, it can be of value in improving their well-being and overall quality of life. In these cases, surgical intervention may be more frequently indicated than in tumors with an osteoblastic or mixed pattern.

### Introduction

Soft tissue sarcomas have a potential for distant metastasis, and a significant proportion of cases are resistant to recent multimodal treatments involving chemotherapy, radiotherapy and wide local resection.[1,2] Lung metastasis appears to be most common in patients with distant tumor recurrence, is life threatening, and from recent reports describing increased survival, may be best countered through aggressive metastasectomy.[3,4]

In contrast, skeletal metastasis occurs with a much lower incidence when compared with the lung,[5,6] but may cause some orthopedic problems. Metastatic lesions from soft tissue sarcomas are characterized by osteolytic destruction with a permeative or moth-eaten pattern.[7] They increase the risk of pathological or impending fracture and instability of the spine which may in turn lead to intractable pain and disfunction.[7,8] Almost half of the metastatic bony lesions sustained pathological fracture in the previous study.[7] Therefore, appropriate surgical treatments for metastatic bony lesions must be considered for improving the patient's quality of life, and possibly prolonging their survival.

Apart from a couple of sporadic cases,[8–10] there have been few studies undertaken to analyse the outcome of surgical treatment for skeletal metastasis from soft tissue sarcomas. In the present report, the authors describe the results of aggressive surgeries, including wide local resection with prosthetic replacement, to deal with these cases.

### Subjects and methods

Thirty-two patients with skeletal metastasis were identified from 302 cases of soft-tissue sarcomas at the authors' institutions over a 20-year period (1975–1996). The patients' primary lesions were removed with no evidence of recurrence. Skeletal metastasis was diagnosed by radiological findings, including a bone scan. Direct invasion to local

Correspondence to: H. Yoshikawa, Department of Orthopaedic Surgery, Osaka Medical Center for Cancer and Cardiovascular Diseases, 1–3–3, Nakamichi, Higashinari-ku, Osaka 537, Japan. Tel. +81 6 9721181; Fax. +81 6 9730216; E-mail: ic7h-yskw@asahi-net.or.jp

1357-714X/98/020107–08 ©1998 Carfax Publishing Ltd bones from the primary soft tissue sarcomas was excluded from skeletal metastasis. Twenty of the 32 patients with bone metastasis received surgical treatments for the metastatic lesion, and three of the 20 patients sustained an additional surgery for another metastatic lesion on the same day or at a later time point. All lesions were confirmed histologically as metastasis from the original sarcoma after surgery. Surgery was not indicated for the remaining 12 patients because their general condition was too poor due to metastases to other important organs, or because the skeletal metastases were too disseminated to be addressed by surgical intervention. The clinical characteristics of the patients who underwent surgical treatment for skeletal metastases are summarized in Table 1. The fracture status of the metastatic lesions is also included: impending fracture in seven lesions, pathological fracture in 13 lesions, and no fracture in three lesions. Surgery was indicated based on the following parameters: life expectancy subjectively estimated to be more than 1 month, and evidence of: (1) solitary bone metastasis without any other metastasis, or with minimal metastases in other organs; (2) bone metastases, solitary or multiple, with pathological fracture or impending fracture; or (3) bone metastases, solitary or multiple, causing neurological symptoms such as paresis and intractable pain.

Twenty lesions from 19 patients presented with pain due to the metastatic lesion, but three lesions (the sacrum in case 19, the rib in case 11 and the humerus in case 20) were pain free and detected radiologically. The surgical procedures for 23 metastatic bony lesions were as follows: wide resection with prosthetic replacement in five lesions; wide or marginal resection without reconstruction in four lesions; intramedullarly nailing with curettage and methylmethacrylate cementation in four lesions; marginal resection of vertebral body with replacement by a ceramic prosthesis[11] in three lesions; laminectomy in three lesions; intramedullarly nailing in two lesions; and curettage in two lesions. A periacetabular lesion was reconstructed by a newly designed prosthesis with a constrained joint mechanism.[12]

In 13 patients (cases 1–4, 6, 9–12, 14, 18, 19 and 20) surgery was undertaken to remove the metastatic tumor with the expectation of prolonging the patient's survival. Laminectomy was performed as an emergency surgery in three patients (cases 15, 16 and 17) to prevent paralysis, and followed by postoperative radiotherapy (40–50 Gy). Palliative curettage was performed for the huge iliac mass (case 13) which caused intractable pain due to severe compression of the femoral nerve. In the remaining three cases (cases 5, 7 and 8), the operations were performed palliatively to relieve pain and ease nursing care.

Pain relief for 17 patients with pain prior to surgery, and walking ability in 10 patients with surgery of the lower extremity and the periacetabular region, were evaluated according to the criteria of Enneking *et al.*[13] The follow-up period ranged from 5 to 85 months (mean, 36.3 months) after the date of the first admission. Survival was calculated according to the methods of Kaplan and Meier.[14] Survival points were based on survival after the first surgery for a metastatic bony lesion.

### Results

### *Local control*

Local recurrence after tumor resection at the wide or marginal margin was not detected clinically during follow-up period. Mild wound infections developed in two patients, both of which resolved with antibiotic therapy. No major complications, such as deep infection, were observed. There were no instances of prosthetic dislocation, loosening, or failure. Fig. 1 and Fig. 2 show the representative cases that received wide local resection and prosthetic replacement for the solitary metastatic lesion.

### *Pain relief and function*

Fifteen out of 19 patients with pain prior to surgery achieved relief of pain, restored mobility and the use of affected limbs within 1 week after surgery: no medication was used for 10 patients; non-narcotic medication was used only occasionally for five patients. They demonstrated improvement compared with their preoperative pain status, and increased independence during activity. Ease of care was enhanced and pain relief was achieved, even in non-ambulatory patients, in spite of the extent of the disease. The remaining four patients needed intermittent or continuous narcotics after surgery; one patient (case 5) failed to be stabilized with internal fixation due to rapid destruction of the lesion, and three patients achieved little pain relief after laminectomy. Relief from pain was achieved in all the patients whose treatment involved tumor resection and prosthetic replacement for their spinal metastases (Fig. 3).

In terms of ambulatory status, two were able to walk without any supports after surgery, and four could walk with a cane, two could walk with crutches, and two required a wheelchair. Because of the palliative mass reduction of the huge iliac metastasis (case 13), intractable pain due to compression of the femoral nerve disappeared immediately after surgery, and the patients were able to walk for 6 months until their condition deteriorated (Fig. 4). In three cases, laminectomy reduced and temporarily prevented paralysis, but resulted in a complete paraplegia after 2–4 weeks due to tumor progression. Tumor resection and prosthetic replacement for spinal metastasis pre-

**Table 1**. *Clinical data on patients who underwent surgical management for skeletal metastases from soft tissue sarcomas*

| No. | Age | Sex | Primary site | Diagnosis* | Metastatic lesion (fracture status)**/surgical procedure | Results and status*** |
|---|---|---|---|---|---|---|
| 1 | 78 | F | Thigh | MFH | Femur (I)/curettage + internal fixation + cementation | DID, 56 (12) |
| 2 | 28 | F | Thigh | ASPS | Femur (I)/wide resection + prosthesis | DID, 5 (4) |
| 3 | 25 | F | Thigh | ASPS | Femur (I)/curettage + internal fixation + cementation | DOD, 48 (37) |
| 4 | 37 | F | Thigh | ASPS | Femur (I)/curettage + cementation | DOD, 41 (21) |
| 5 | 13 | F | Buttock | RMS | Femur (F)/internal fixation | DOD, 15 (5) |
| 6 | 26 | M | Upper arm | M.schwan | Femur (F)/wide resection + prosthesis | DOD, 28 (5) |
| 7 | 52 | M | Foot | Synovial | Tibia(I)/curretage + internal fixation + cementation | DOD, 17 (16) |
| 8 | 37 | M | Forearm | Synovial | Tibia (F)/currettage + internal fixation + cementation | DOD, 41 (5) |
| 9 | 62 | F | Chest wall | MFH | Humerus (I)/wide resection + prosthesis | NED, 17 (7) |
| 10 | 36 | M | Duodenum | LMS | Scapula (F)/wide resection | DOD, 64 (38) |
| 11 | 62 | F | Thigh | LMS | Rib (N)/wide resection | DOD, 52 (28) |
| 12 | 67 | M | Knee | MFH | Ilium (F)/wide resection + prosthesis | DOD, 33 (14) |
| 13 | 65 | M | Stomach | LMS | Ilium (F)/currettage | DOD, 20 (8) |
| 14 | 34 | M | Popliteal | Synovial | C5, 6 (F)/marginal resection + prosthesis | DOD, 28 (15) |
| 15 | 12 | M | Retroperitoneum | RMS | Th 12 (F)/laminectomy | DOD, 11 (9) |
| 16 | 71 | F | Knee | MFH | L3 (F)/laminectomy | DOD, 35 (5) |
| 17 | 8 | F | Thigh | RMS | L4 (F)/laminectomy | DOD, 15 (7) |
| 18 | 48 | M | Thigh | MFH | Th3 (F)/resection + prosthesis, scapula (F)/marginal resection | DOD, 85 (19) |
| 19 | 26 | F | Buttock | ASPS | Sacrum (N)/wide resection, femur (I)/wide resection + prosthesis | DOD, 71 (57) |
| 20 | 47 | F | Leg | M.schwan | Humerus (N)/internal fixation, L5 (F)/marginal resection + prosthesis | DOD, 44 (16) |

*MFH, malignant fibrous histiocytoma; ASPS, alveolar soft-part sarcoma; Synovial, synovial sarcoma; LMS, leiomyosarcoma; RMS, rhabdomyosarcoma; M.schwan, malignant schwannoma.

**I, impending fracture; F, fracture; N, no fracture.

***Prognosis of the patients: DOD, dead of disease; DID, dead of intercurrent disease; AWD, alive with disease; NED, no evidence of disease; number, months from initial admission; number in parentheses, months from surgery for skeletal metastasis.

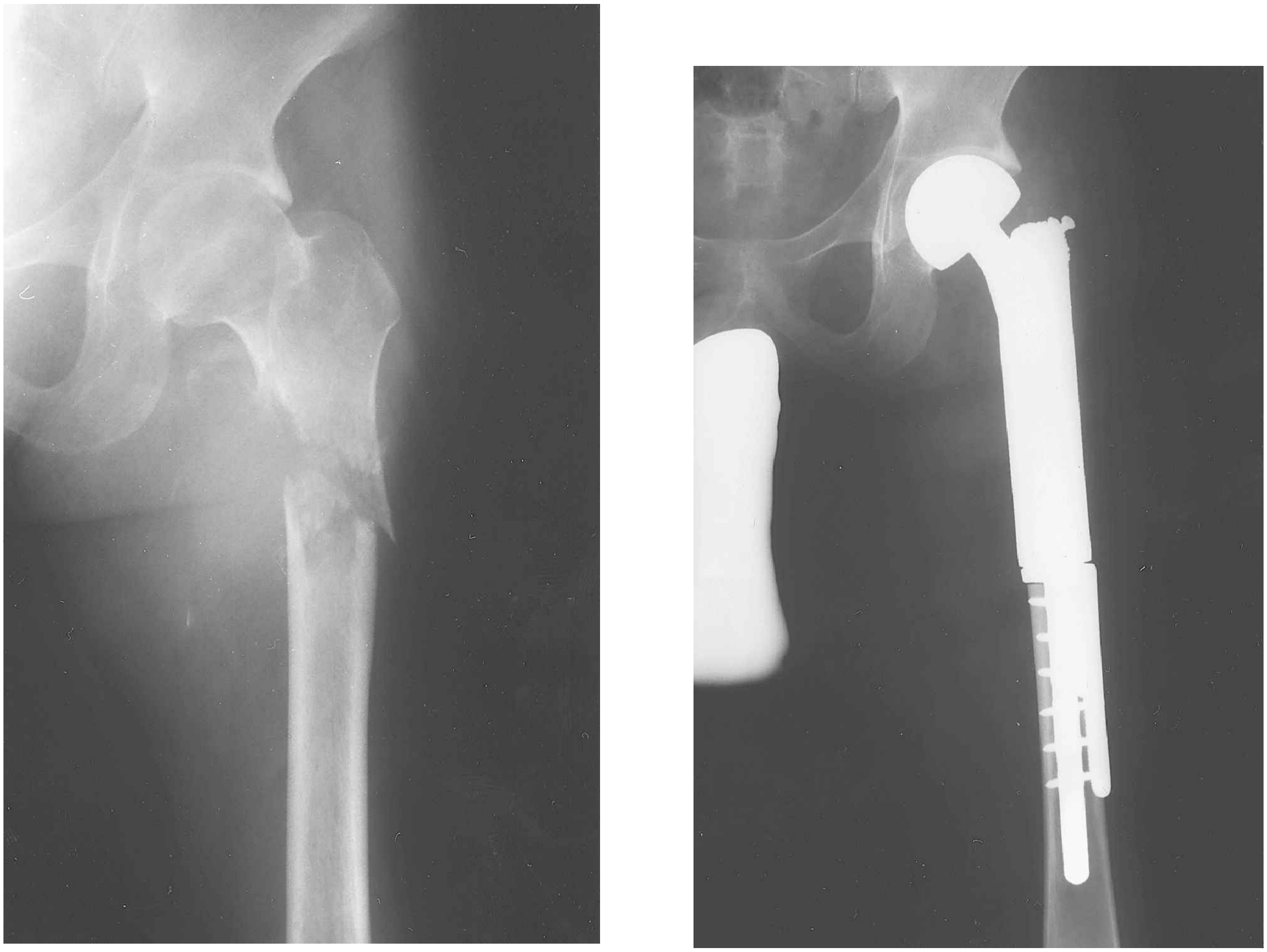

**Fig. 1.** *(Left) A 26-year-old male with metastasis of malignant schwannoma to the femur. An osteolytic lesion with a pathological fracture is discernible. (Right) The patient was ambulatory after wide resection and prosthetic replacement (Kotz system) for 4 months until his illness reached a terminal condition.*

vented neurological dysfunction during the follow-up periods in these patients.

*Survival*

The overall mean survival period for the 20 patients who received surgical treatment, calculated from surgery for bone metastasis, was 13.4 months (range, 4–57 months). Of these 20 patients, 17 died of diseases with a mean survival period of 17.9 months (range, 5–57 months) from the time of surgery, 14 died of pulmonary metastases, and three (cases 15, 17 and 19) died of disseminated bone metastases. Two patients (cases 1 and 2) died of other causes, with survivals of 12 and 4 months from surgery for bone metastases, and only one patient is now alive without any metastasis 7 months after surgery. The overall estimated survival for patients with surgical treatment for bone metastasis from sarcomas of soft tissue are shown in Fig. 5. One and 2-year survivals from surgery for bone metastasis were 62.4% and 24.9%, respectively. No 5-year survivors were recorded. On the other hand, the remaining 12 patients, for whom surgery was not indicated, died with a mean survival period of 3.5 months (range, 1–5 months).

### Discussion

Skeletal metastasis has been considered as a hematogenous dissemination and a terminal stage in the lives of sarcoma patients.[2,5,6,15] Because of this poor prognosis, metastatic bony lesions are not always managed aggressively. However, advances in the oncological management of patients with disseminated cancer have led to increased survival and quality of life.[16] Moreover, much experience has been gathered from the field of limb-saving procedures in primary tumors of the musculoskeletal system, and radical local excision and endoprosthetic replacement have been used commonly for the management of isolated skeletal metastases from cancers.[8,9,12,17] Therefore, orthopedic management for patients with skeletal metastasis from sarcomas must be also encouraged, even if their survival periods are limited. Despite the palliative nature of the treatment, an aggressive program of fracture management for long bones utilizing current techniques

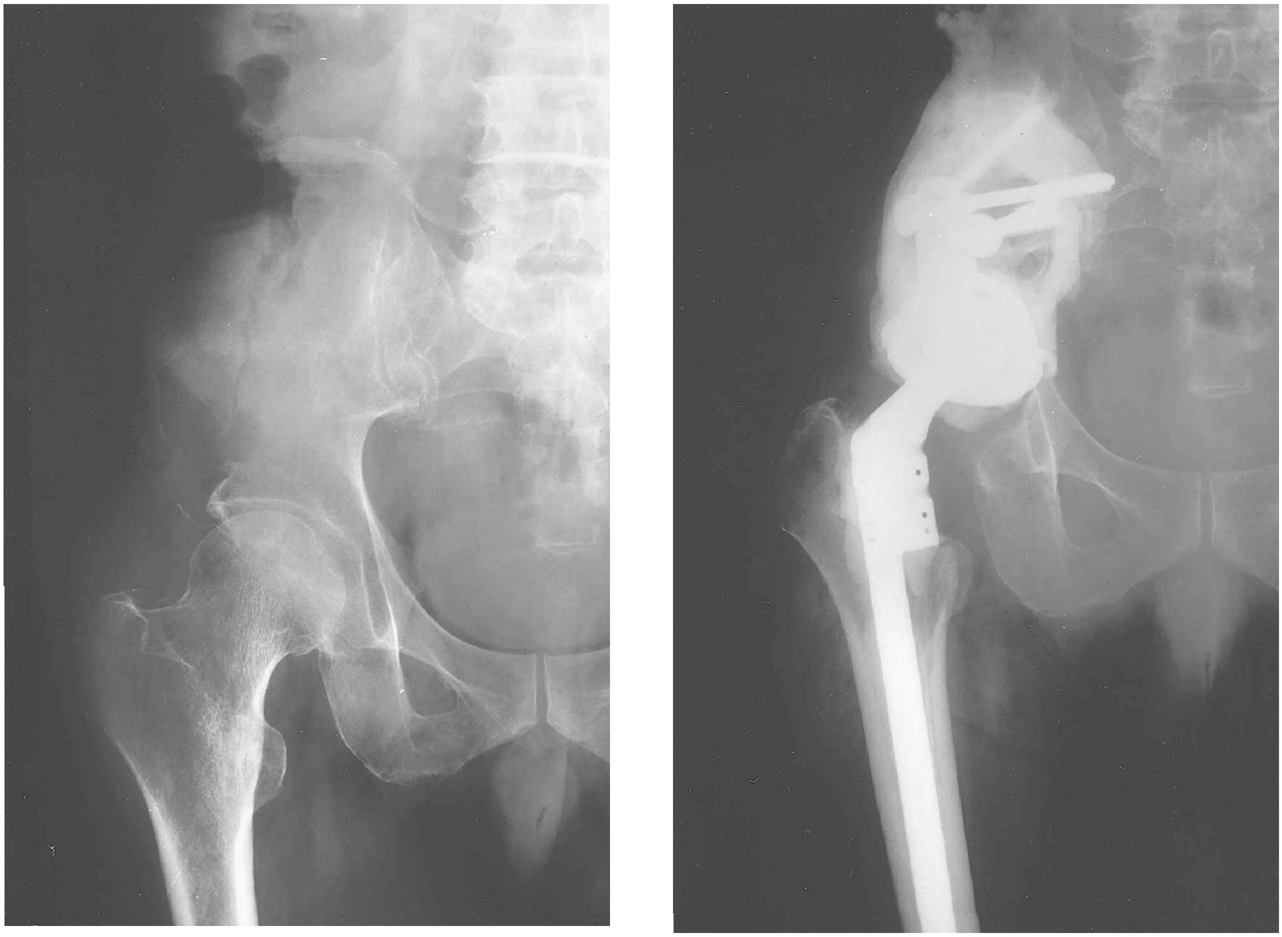

**Fig. 2.** *(Left) A 67-year-old male with metastasis of malignant fibrous histiocytoma of the knee to the acetabulum. An osteolytic lesion with a pathological fracture is discernible. (Right) The patient was ambulatory after wide resection and prosthetic replacement for 12 months until his illness reached a terminal condition.*

with internal fixation to provide secure stabilization, or prosthetic replacement to restore joint function, provides considerable benefits. From this perspective, a diffuse involvement of skeleton by soft tissue sarcomas may not be a contraindication to surgical treatment.

In the present study, methylmethacrylate was used in five cases for the internal fixation of pathological fractures, a procedure that has been used widely in dealing with metastasis from carcinoma.[18,19] This procedure afforded immediate stability and enabled the patients to resume walking with good relief of pain in all cases. Since metastasis from soft tissue sarcomas leads almost exclusively to osteolytic lesions,[7] stabilization with methylmethacrylate appears to be a successful strategy for countering this possibility. Recent advances in prostheses have led to endoprosthetic replacement as an option for the treatment of metastatic bony lesions in addition to primary bone tumors.[17,20] The use of an endoprosthesis permits tumor resection and rapid stabilization in a single surgical event. In all of the five cases involving the lower extremity of a limb where this procedure was employed, pain relief was achieved and the ambulatory status was enhanced immediately following surgery. Since a local recurrence has not been detectable during the follow-up period, stabilization via endoprosthetic replacement appears to work well for the treatment of lesions metastasizing to the proximal femur and humerus from soft tissue sarcomas. By using internal fixation with methylmethacrylate or endoprosthetic replacement, pain relief was achieved satisfactorily in most cases, and considerable function was gained in the cases involving a lower limb extremity. Therefore, the surgical procedures employed for dealing with metastases from carcinomas are also applicable for skeletal metastasis from soft tissue sarcomas.

The spine is the most common site of skeletal metastasis in soft-tissue sarcomas,[7] as well as in most carcinomas, most likely via Batson's vertebral vein system,[21] raising the possibility of surgical management as a treatment option. Laminectomy and postoperative radiotherapy were performed on an emergency basis in only three patients. This palliative treatment prevented paralysis temporally but could not relieve pain due to spinal instability. However, in three cases who received tumor resection and prosthetic replacement for their spinal lesion, pain relief was achieved because of spinal cord

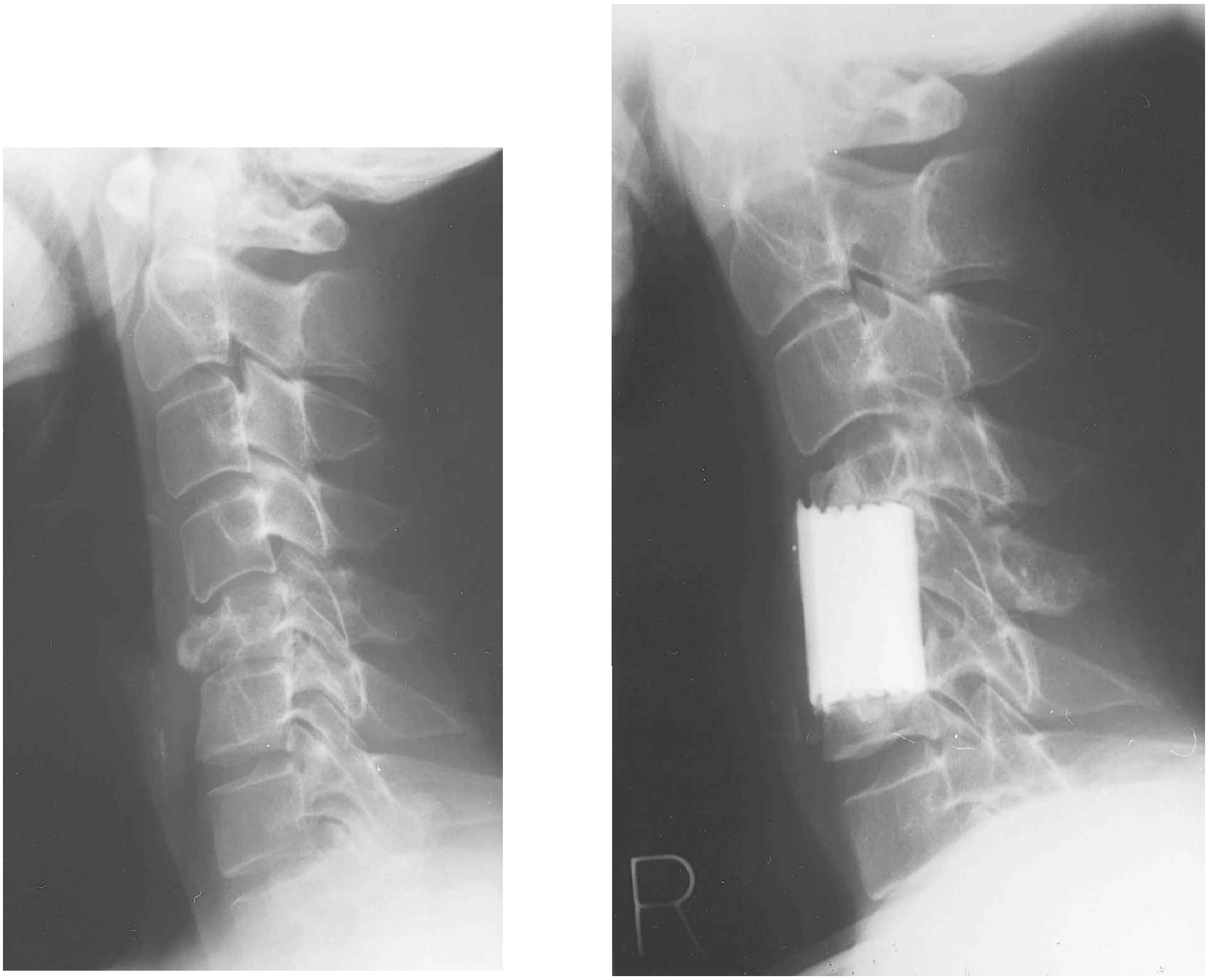

**Fig. 3.** *(Left) A 34-year-old man had severe neck pain, resisting any analgesics because of C5 metastasis from synovial sarcoma of the popliteal region. A destructive metastasis with compression fracture of the C5 body is discernible. (Right) The affected vertebra was resected and replaced with a ceramic prosthesis. The severe neck pain was relieved after surgery.*

decompression and restoration of spinal stability, and survival after surgery extended beyond 1 year (mean, 17 months). Although radical resection of the lesions in the spine is impossible, mass reduction of the tumor may ensure prevention of local recurrence for a long period, and increase the efficacy of radiation in eradicating microscopic foci of the tumor around the spinal cord.[11] Therefore, if surgical treatment is indicated, both decompression of the neural tissues by tumor resection and restoration of spinal stability, in spite of their aggressiveness, seem to be essential to restore neurological function, control pain, and provide patients with improved mobility and quality of life. Prosthetic replacement surgery, with which decompression and stabilization can be achieved simultaneously, is an excellent choice for vertebral body metastasis.

In terms of prognosis, mean survival from surgery for bone metastasis is 13.4 months for 20 patients, which is similar to the findings of previous reports describing the clinical outcomes in cases of skeletal metastases from carcinomas; for example, 5.6 months in patients who received endoprosthetic replacement for proximal femur,[20] and 10 months in patients who received intramedullarly nailing and cementation of the humerus,[19] and 15.4 months in patients with internal fixation of a pathological fracture.[18] Of particular note in our study is that in three of the four alveolar soft tissue sarcomas, the mean survival period following surgery was more than 3 years, indicating that surgical intervention seems well indicated for this particular tumor, and longer survival might be expected in other cases, for example, skeletal metastases from thyroid cancer.[16] Unfortunately, in spite of aggressive surgical treatment, it is disappointing that no 5-year survivor after surgery was recorded, although cases of a greater than 5-year survival period after metastasectomy for lung or lymphnode metastases have been reported.[4,22] While somewhat speculative, it is reasonable to conclude that metastases to the skeleton may be considered to be a more significant indicator of a poor outcome when contrasted with lung or lymphnode for patients with soft tissue sarcomas.

In summary, surgical treatment for skeletal metastases from soft-tissue sarcomas cannot save the life

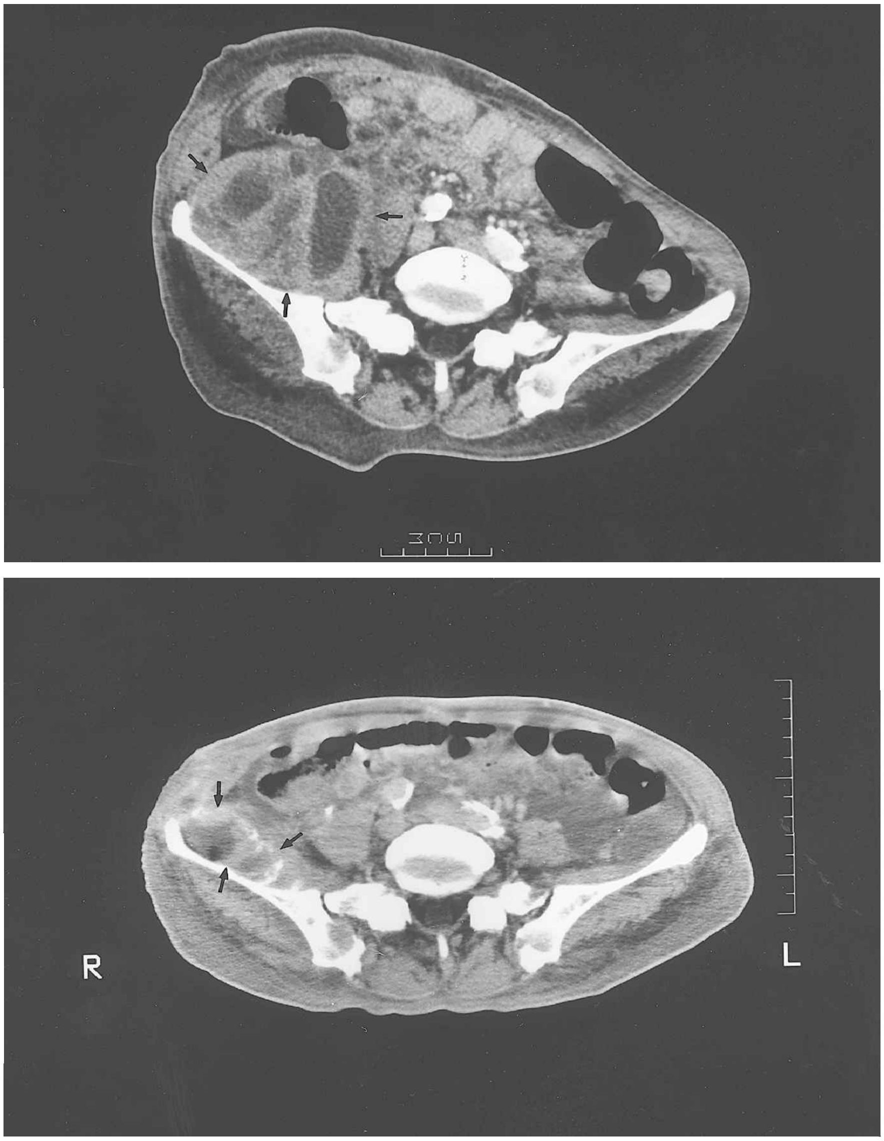

**Fig. 4.** *(Top) A 65-year-old man had severe radiated pain of the right lower extremity and severe restriction of extension of the right hip, because of a huge metastatic lesion (arrows) of the ilium from leiomyosarcoma of the stomach. (Bottom) The radiated pain was relieved immediately after a palliative curettage, and the patient could walk for 6 months until his illness reached a terminal condition. A CT scan 1 month after surgery shows only a residual cystic lesion (arrows).*

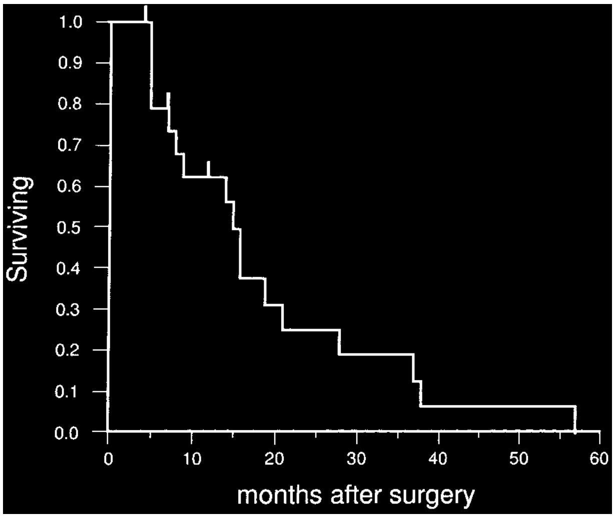

**Fig. 5.** *Overall estimated survival for patients with surgical treatment for skeletal metastasis from soft tissue sarcomas. Survival calculated from time of surgery for metastatic bony lesion.*

of the patient, but may be of value in improving their well-being and overall quality of life. We believe that, in these cases, surgical intervention may be more frequently required than for metastatic tumors with an osteoblastic or mixed pattern, such as prostatic or breast carcinomas. Further studies will be required to confirm that surgical intervention in cases of skeletal metastasis from soft tissue sarcomas leads to improvements in patient survival.

### Acknowledgements

The authors thank Dr. David C. Morris for critical reading of the manuscript. This study was supported in part by a grant from the Japanese Ministry of Health and Welfare.

### References

1 Gustafson P. Soft tissue sarcoma. Epidemiology and prognosis in 508 patients. *Acta Orthop Scand* 1994; 65:1–29.
2 Enzinger FM, Weiss SW. *Soft Tissue Tumors*, 3rd ed. St. Louis: Mosby-Year Book, Inc, 1995: 1–38.
3 Putnam JB, Roth JA, Wesley MN, *et al.* Analysis of prognostic factors in patients undergoing resection of pulmonary metastases from soft tissue sarcomas. *J Thorac Cardiovasc Surg* 1984; 87:260–8.
4 Ueda T, Uchida A, Kodama K, *et al.* Aggressive pulmonary metastasectomy for soft tissue sarcomas. *Cancer* 1993; 72:1991–25.
5 Huth JF, Eilber FR. Patterns of metastatic spread following resection of extremity soft-tissue sarcomas and strategies for treatment. *Semin Surg Oncol* 1988; 4:20–6.
6 Potter DA, Kinsella T, Glatstein E, *et al.* High-grade soft tissue sarcomas of the extremities. *Cancer* 1986; 58:190–205.
7 Yoshikawa H, Ueda T, Mori S, *et al.* Skeletal metastasis from soft-tissue sarcomas: incidence, patterns, and radiologic features. *J Bone Joint Surg* 1997; 79(B): 548–52.
8 Edmonson JH, Sim FH, Gunderson LL, *et al.* Bone and soft-tissue sarcoma. In: Sim FH, ed. *Diagnosis and management of metastatic bone disease. A multidisciplinary approach*. New York: Raven Press, 1988:19–33.
9 Yazawa Y, Frassica FJ, Chao EYS, *et al.* Metastatic bone disease. A study of the surgical treatment of 166 pathologic humeral and femoral fractures. *Clin Orthop* 1990; 251:213–9.
10 Gaffen EV, Wobbes T, Veth RPH, *et al.* Operative management of impending pathological fractures: A critical analysis of therapy. *J Surg Oncol* 1997; 64:190–4.
11 Hosono N, Yonenobu K, Fuji T, *et al.* Orthopaedic management of spinal metastases. *Clin Orthop* 1995; 312:148–59.
12 Uchida A, Myoui A, Araki N, *et al.* Prosthetic reconstruction for periacetabular malignant tumors. *Clin Orthop* 1996; 326:238–45.
13 Enneking WF, Dunham W, Gebhardt MC, *et al.* A system for the functional evaluation of reconstructive procedures after surgical treatment of tumors of the musculoskeletal system. *Clin Orthop* 1993; 286:241–6.
14 Kaplan EL, Meier P. Non-parametric estimation from incomplete observations. *J Am Stat Assoc* 1958; 53:457–81.
15 Potter DA, Glenn J, Kinsella T, *et al.* Patterns of recurrence in patients with high grade soft-tissue sarcomas. *J Clin Oncol* 1985; 3:353–66.
16 Galasko CSB. *Skeletal Metastases*. London: Butterworths & Co Ltd, 1986.
17 Harrington KD. Orthopaedic management of extremity and pelvic lesions. *Clin Orthop* 1995; 312;136–47.
18 Harrington KD, Sim FH, Enis JE, *et al.* Methylmethacrylate as an adjunct in internal fixation of pathological fractures. *J Bone Joint Surg* 1976; 58(A): 1047–55.
19 Lewallen RP, Pritchard DJ, Sim FH. Treatment of pathologic fractures or impending fractures of the humerus with rush rods and methylmethacrylate. Experience with 55 cases in 54 patients, 1968–1977. *Clin Orthop* 1982; 166:193–8.
20 Lane JM, Sculco TP, Zolan S. Treatment of pathological fractures of the hip by endoprosthetic replacement. *J Bone Joint Surg* 1980; 62(A):954–9.
21 Batson OV. The vertebral vein system. *Am J Roentgenol* 1957; 78:195–212.
22 Fong Y, Coit DG, Woodruff JG, *et al.* Lymph node metastasis from soft tissue sarcoma in adults. Analysis of data from a prospective database of 1772 sarcoma patients. *Ann Surg* 1993; 217:72–7.
